# The role of mobility and health disparities on the transmission dynamics of Tuberculosis

**DOI:** 10.1186/s12976-017-0049-6

**Published:** 2017-01-28

**Authors:** Victor Moreno, Baltazar Espinoza, Kamal Barley, Marlio Paredes, Derdei Bichara, Anuj Mubayi, Carlos Castillo-Chavez

**Affiliations:** 1 Simon A. Levin Mathematical, Computational and Modeling Sciences Center, Arizona State University, Tempe, AZ US; 2 School of Human Evolution and Social Change, Arizona State University, Tempe, AZ US; 3 Department of Mathematical Sciences, University of Cincinnati, Cincinnati, OH USA; 4 Department of Mathematics and Physics, University of Puerto Rico, Cayey, PR USA; 5 Department of Mathematics & Center for Computational and Applied Mathematics, California State University, Fullerton, CA USA; 6 Rector’s Office, Yachay Tech University, Urcuqui, Ecuador

**Keywords:** Tuberculosis, Residence times, Heterogeneity, Exogenous re-infection, Direct first time infection rate, Metapopulation model

## Abstract

**Background:**

The transmission dynamics of Tuberculosis (TB) involve complex epidemiological and socio-economical interactions between individuals living in highly distinct regional conditions. The level of exogenous reinfection and first time infection rates within high-incidence settings may influence the impact of control programs on TB prevalence. The impact that effective population size and the distribution of individuals’ residence times in different patches have on TB transmission and control are studied using selected scenarios where risk is defined by the estimated or perceive first time infection and/or exogenous re-infection rates.

**Methods:**

This study aims at enhancing the understanding of TB dynamics, within simplified, two patch, risk-defined environments, in the presence of short term mobility and variations in reinfection and infection rates via a mathematical model. The modeling framework captures the role of individuals’ ‘daily’ dynamics within and between places of residency, work or business via the average proportion of time spent in residence and as visitors to TB-risk environments (patches). As a result, the effective population size of Patch i (home of i-residents) at time t must account for visitors and residents of Patch i, at time t.

**Results:**

The study identifies critical social behaviors mechanisms that can facilitate or eliminate TB infection in vulnerable populations. The results suggest that short-term mobility between heterogeneous patches contributes to significant overall increases in TB prevalence when risk is considered only in terms of direct new infection transmission, compared to the effect of exogenous reinfection. Although, the role of exogenous reinfection increases the risk that come from large movement of individuals, due to catastrophes or conflict, to TB-free areas.

**Conclusions:**

The study highlights that allowing infected individuals to move from high to low TB prevalence areas (for example via the sharing of treatment and isolation facilities) may lead to a reduction in the total TB prevalence in the overall population. The higher the population size heterogeneity between distinct risk patches, the larger the benefit (low overall prevalence) under the same “traveling” patterns. Policies need to account for population specific factors (such as risks that are inherent with high levels of migration, local and regional mobility patterns, and first time infection rates) in order to be long lasting, effective and results in low number of drug resistant cases.

## Background

Tuberculosis (TB), a communicable disease caused by bacteria (*Mycobacterium tuberculosis*), remains among one of the leading causes of death worldwide. According to the World Health Organization’s (WHO) report, 9.6 million people developed symptomatic TB infections resulting in 1.5 million TB-associated deaths in 2014 [1]. Despite the existence of treatment and vaccine, it is estimated that one-third of the world population serves as TB reservoirs. The majority of these latently infected individuals live in developing countries where they are exposed to multiple TB risk factors. Individuals living in rural areas, mainly in developing countries, and in general below the poverty line, disproportionately contribute to the documented TB burden [2, 3]. Data has shown strong association between poverty and TB, primarily in economically underprivileged countries [4]. Vulnerable groups are at greater risk of TB infection compared with the general population because of overcrowding of individuals and substandard living. Poor working conditions, poor nutrition, inter-current diseases, and migration from (or to) higher-risk communities (or nations) are other known risk factors for TB [3]. The Worldwide TB incidence rates seemed to have peaked (2004) after the HIV epidemic (1997) and then decreased at a rate of less than 1% per year. Nonetheless, the overall worldwide TB-burden continues to rise as the world population continues to grow rapidly [5]. In addition, inappropriate treatment and the use of poor quality drugs have led to wild and antibiotic resistant strains contributing to the already high levels of TB-active incidence in recent years making TB a major global public health threat.

Gomes et al. [6] found that TB-reinfection rates, that is, reinfection after successful treatment, are higher than TB infection rates among those with no prior TB-experience. In their model, they propose two mechanisms (for ongoing high prevalence in some regions): (i) past infections increase susceptibility to reinfection (ii) differences in susceptibility to infection contribute to increased re-infection rates among the treated. The study of these possibilities suggests that the last mechanism may be better supported by data. Consequently, Gomes et al. [6] noted that, the rates of reinfection are higher at the population level than at the individual level.

Metapopulation type transmission models [7–10] offer a powerful set up for the study of the dynamics of TB infected individuals, on which the effectiveness of population-level TB interventions like treatment, movement restrictions, and local control measures can be studied. Models in [11, 12] offer a set up aimed at exploring the impact of a mobile populations in a n-patch system with risk heterogeneity in which individuals immigrated between different risk environments. However, these models made use of an Eulerian approach for mobility where the concepts of residence times and effective population size were not incorporated; an approach that, for example does not allow for the identification of the place of residency of treated or quarantined individuals as well as the impact of effective population size on transmission. Prior TB-related studies have estimated TB incidence growth rates, explored the impact of interventions aimed at reducing TB prevalence and the impact of exogenous reinfection on TB dynamics, however, movement of individuals that keep track of place of resident have been in general ignored (see [10]).

Limited TB studies have considered models incorporating movement via mass transportation within a Lagrangian approach based on budgeting contacts as a function of residency times (see [10]), or taking into account the impact of sudden blips of immigration, which may be central to TB re-emergence [13–17], or that account for co- infections, specially with HIV [18–23], or that account for relapse [6, 24–27], or that account for antibiotic, drug, and ultra-drug resistance [28–33], or models that account for TB re-activation and progression [34–36]. In addition, models assuming negligible immigration might not capture the real dynamics of tuberculosis in open populations when high levels of diversity is caused by immigrants [29].

Research aimed at increasing the understanding of the transmission dynamics of TB that explicitly incorporate the role of heterogeneous TB-risk environments is limited. The goal of this study is to understand the impact of residence times and population sizes, across distinct risk environments, on the TB transmission dynamics when risk being defined in terms of new infection and/or exogenous infection rates. We define residence time in a place, as the average proportion of daily time an individual spends in a given region or patch. In particular, we address three questions (i) *How does mobility changes TB prevalence via the trade-off between exogenous and direct first time infection rates?*, (ii) *How differences in TB prevalence and population sizes in the patches can influence the impact of mobility on the total number of infections?* and (iii) *Which among the two, direct first time infection rates and exogenous re-infection rates, is capable of sustaining higher TB prevalence?*

## Methods

We consider a model for the transmission dynamics of TB in populations interacting in two distinct regions/patches. First, we introduce a model with one patch and then extend it to capture two patches by explicitly incorporating short term movement of individuals between and within patches. The two-patch mobility model is used to address the role of movement and patch-risk on TB dynamics. Relevant definition of concepts (or nomenclature) and case studies (or numerical scenarios) that are used here to achieve goals are collected in Table 1.

**Table 1:** Definitions and scenarios in the study

Nomenclature	
Risk	Interpreted based on levels of infection rate, prevalence,
	or average contacts (via population size)
High-risk patch	Defined either by high direct first time infection rate (i.e., high *β*
	which leads to high corresponding $\mathcal {R}_{0}$) or by high exogenous re-infection rate
	(i.e., high *δ*)
Enhanced socio-economic conditions (reducing health disparity)	Defined by better healthcare infrastructure which is incorporated by high prevalence of a disease (i.e., high *I*(0)/*N*) in a large population (i.e., large *N*)
Mobility	Captured by average residence times of an individual in different patches (i.e., by using $\mathbb {P}$ matrix)
Scenarios (assume high-risk and enhanced socio-economic conditions in Patch 1 as compared to Patch 2)	
Scenario 1	$\underbrace {\beta _{1} > \beta _{2}, \ \delta _{1} = \delta _{2}}_{\text {high risk}}$; $\underbrace {\frac {I_{1}(0)}{N_{1}} > \frac {I_{2}(0)}{N_{2}}, \ N_{1} > N_{2};}_{\text {enhanced socio-economic conditions}}$ $\underbrace {\text {vary} \ p_{12} \ \text {when} \ p_{21}\approx 0}_{\text {mobility}}$
Scenario 2	$\underbrace {\beta _{1} = \beta _{2}, \ \delta _{1} > \delta _{2}}_{\text {high risk}}$; $\underbrace {\frac {I_{1}(0)}{N_{1}} > \frac {I_{2}(0)}{N_{2}}, \ N_{1} > N_{2};}_{\text {enhanced socio-economic conditions}}$ $\underbrace {\text {vary} \ p_{12}\ \text {when} \ p_{21}\approx 0}_{\text {mobility}}$

### A simple TB model for one patch with homogenously mixing population

The transmission dynamics of TB in homogeneously mixing populations is represented by systems of differential equations describing the TB contagion. The population in the model is divided into three sub-populations each corresponding to an epidemiological TB state: susceptible individuals (*S*), noninfectious infected, that is, latent individuals (*L*), and actively infectious individuals (*I*).

The model considers two contagion pathways: direct progression (fast dynamics) and endogenous reactivation (slow progression, often years after infection). Susceptible individuals (*S*) may get infected through contacts with individuals with active-infections (*I*), moving to either the noninfectious latent class (*L*) or the actively infectious (*I*) state. The fraction (1−*q*) denotes the proportion of infected individuals that move directly into the infectious stage (*I*). Reactivation from longstanding latent infections is modeled by the transition of individuals from the noninfectious to the infectious state (progression to active TB) via endogenous reactivation (at the per capita rate *γ*), or via exogenous reinfection. Infectious individuals may be treated at the per capita rate *ρ* moving into the non-infectious infected category *L* as total *mycobacterium* elimination is assumed to be non possible.

**Fig. 1:**
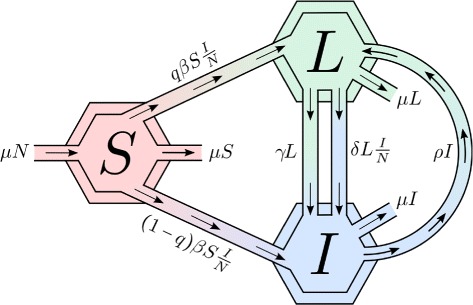
Flow diagram for the single patch three compartment model: susceptible (*S*), infected latent (*L*) and infectious (*I*)

The model assumes that (1) the population is constant; (2) TB-induced deaths are negligible and hence ignored; (3) a fraction of individuals are infectious; (4) individuals may control an active infection without treatment moving back to the latent class; (5) individuals in the latent class may relapse and develop active TB or remain in this class until death due to natural causes (that is, not TB). Figure 1 shows the flow diagram associated with the transmission dynamics of the TB model used.

This model follows the structure used in [34, 37, 38] where exogenous reinfection, fast and slow progression are considered. The basic reproduction number and the existence of a parameters’ range for which there are two stable equilibria, disease free and endemic steady states are highlighted in [34, 37, 38]. The basic reproduction number of the model is given by

1 $$ \mathcal R_{0}=\frac{\beta (\gamma+(1-q)\mu)}{\mu(\mu+\rho+\gamma)}  $$

#### Interpretation of the $\mathcal {R}_{0}$ in terms of the parameters needs to go here.

The basic reproduction number ($\mathcal R_{0} $) gives the average number of secondary infections generated by a typically infected individual in a population of susceptible individuals. In the presence of exogenous reinfection, excluding fast progression (*q*=1 and *δ*>0), it is known that the model can support two stable equilibria [34]. The role of TB, in this case would be closely linked not only to $\mathcal {R}_{0}$ but also to the initial conditions. We proceed to build a two-patch model, under a residency-time matrix, using the model outlined above.

### A two-patch TB model with heterogeneity in population through residence times

Let *N*_1_ and *N*_2_ be the host population of Patch 1 and 2, respectively. The population of Patch 1 spends, on the average, the proportion *p*_11_ of its time in residency in Patch 1 and the proportion *p*_12_ of its time in Patch 2 (*p*_11_+*p*_12_=1). Similarly, residents of Patch 2 spend the proportion *p*_22_ of their time in Patch 2 and *p*_21_=1−*p*_22_ in Patch 1. Hence, at time *t*, the *effective population* in Patch 1 is *p*_11_ *N*_1_+*p*_21_ *N*_2_ while the *effective population* of Patch 2, at time *t*, is *p*_12_ *N*_1_+*p*_22_ *N*_2_. The susceptible population of Patch 1 (*S*_1_) may become infected in Patch 1 (if currently in Patch 1, i.e. *p*_11_ *S*_1_) or in Patch 2 (if currently in Patch 2, i.e. *p*_12_ *S*_2_). In short, from this Lagrangian approach to capture movement of individuals, we conclude that the *effective proportion of infectious* individuals in Patch 1 at time *t* is

$$\frac{p_{11}I_{1}+p_{21}I_{2}}{p_{11}N_{1}+p_{21}N_{2}}. $$

Thus, the dynamics of infection among susceptible, resident individuals of Patch 1 is given by

2 $$ \dot S_{1}=\mu_{1}N_{1}-\beta_{1} p_{11}S_{1}\frac{p_{11}I_{1}+p_{21}I_{2}}{p_{11}N_{1}+p_{21}N_{2}}-\beta_{2}p_{12}S_{1}\frac{p_{12}I_{1}+p_{22}I_{2}}{p_{12}N_{1}+p_{22}N_{2}}-\mu_{1} S_{1}.  $$

The dynamics of Patch 1 residents acquiring latent, asymptomatic infections, is,

3 $$ \begin{aligned} \dot{L}_{1}&=q\beta_{1} p_{11}S_{1}\frac{p_{11}I_{1}+p_{21}I_{2}}{p_{11}N_{1}+p_{21}N_{2}}+q\beta_{2}p_{12}S_{1}\frac{p_{12}I_{1}+p_{22}I_{2}}{p_{12}N_{1}+p_{22}N_{2}}\\ & \quad -\delta_{1} p_{11}L_{1}\frac{p_{11}I_{1}+p_{21}I_{2}}{p_{11}N_{1}+p_{21}N_{2}}-\delta_{2}p_{12}L_{1}\frac{p_{12}I_{1}+p_{22}I_{2}}{p_{12}N_{1}+p_{22}N_{2}}-(\gamma_{1}+\mu_{1})L_{1}+ \rho_{1} I_{1}, \end{aligned}  $$

and the dynamics of the Patch 1 residents becoming infectious is

4 $$ \begin{aligned} \dot{I}_{1}&=(1-q)\beta_{1} p_{11}S_{1}\frac{p_{11}I_{1}+p_{21}I_{2}}{p_{11}N_{1}+p_{21}N_{2}}+(1-q)\beta_{2}p_{12}S_{1}\frac{p_{12}I_{1}+p_{22}I_{2}}{p_{12}N_{1}+p_{22}N_{2}}\\ & \quad +\delta_{1} p_{11}L_{1}\frac{p_{11}I_{1}+p_{21}I_{2}}{p_{11}N_{1}+p_{21}N_{2}}+\delta_{2}p_{12}L_{1}\frac{p_{12}I_{1}+p_{22}I_{2}}{p_{12}N_{1}+p_{22}N_{2}}+\gamma_{1} L_{1}-(\mu_{1}+\rho_{1}) I_{1}. \end{aligned}  $$

The use of (2), (3),(4) determines the complete dynamics of TB, in two patches, and it is given by the following System (*i*=1,2):

5 $$  \left\{\begin{array}{llll} \dot S_{i}=\mu_{i}N_{i}-\sum_{j=1}^{2}\beta_{j} p_{ij}S_{i}\frac{\sum_{k=1}^{2}p_{kj}I_{k}}{ \sum_{k=1}^{2}p_{kj}N_{k}}-\mu_{i} S_{i},\\ \dot{L}_{i}=q\sum_{j=1}^{2}\beta_{j} p_{ij}S_{i}\frac{\sum_{k=1}^{2}p_{kj}I_{k}}{ \sum_{k=1}^{2}p_{kj}N_{k}}-\sum_{j=1}^{2}\delta_{j} p_{ij}L_{i}\frac{\sum_{k=1}^{2}p_{kj}I_{k}}{ \sum_{k=1}^{2}p_{kj}N_{k}}-(\gamma_{i}+\mu_{i})L_{i}+\rho_{i} I_{i},\\ \dot{I}_{i}=(1-q)\sum_{j=1}^{2}\beta_{j} p_{ij}S_{i}\frac{\sum_{k=1}^{2}p_{kj}I_{k}}{ \sum_{k=1}^{2}p_{kj}N_{k}}+\sum_{j=1}^{2}\delta_{j} p_{ij}L_{i}\frac{\sum_{k=1}^{2}p_{kj}I_{k}}{ \sum_{k=1}^{2}p_{kj}N_{k}}+\gamma_{i} L_{i}-(\mu_{i}+\rho_{i}) I_{i}. \end{array}\right.  $$

Let *N*_*i*_=*S*_*i*_+*L*_*i*_+*I*_*i*_ the total population of Patch *i*,*i*=1,2. System (5) has the same qualitative dynamics than the following reduced system since the total population is constant:

6 $${} \left\{\begin{array}{llll} \dot{L}_{i}=q\sum_{j=1}^{2}\beta_{j} p_{ij}(N_{i}-L_{i}-I_{i})\frac{\sum_{k=1}^{2}p_{kj}I_{k}}{ \sum_{k=1}^{2}p_{kj}N_{k}}-\sum_{j=1}^{2}\delta_{j} p_{ij}L_{i}\frac{\sum_{k=1}^{2}p_{kj}I_{k}}{ \sum_{k=1}^{2}p_{kj}N_{k}}-(\gamma_{i}+\mu_{i})L_{i}+\rho_{i} I_{i},\\ \dot{I}_{i}=\sum_{j=1}^{2}p_{ij}\left((1-q)\beta_{j} (N_{i}-L_{i}-I_{i})+\delta_{j} L_{i}\frac{}{}\right)\frac{\sum_{k=1}^{2}p_{kj}I_{k}}{ \sum_{k=1}^{2}p_{kj}N_{k}}+\gamma_{i} L_{i} -(\mu_{i}+\rho_{i}) I_{i}. \end{array}\right.  $$

**Fig. 2:**
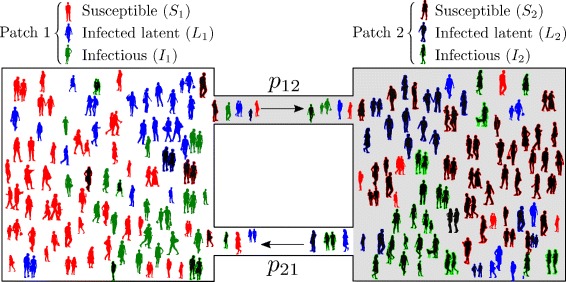
Schematic description of the Lagrangian approach between two patches

A schematic description of the two-patch dynamical model is provided in Fig. 2 and a description of the parameters as well as their estimates from previous studies can be found in Table 2.

**Table 2:** Description of the parameters used in System (6)

Parameters	Description	Ranges(units)	References
*β*_*i*_	Susceptibility to TB invasion in Patch *i*	0.01 - 0.0192 (*y* ^−1^)	[35]
*δ*_*i*_	Susceptibility to exogenous TB progression in Patch *i*	0.0026 - 0.0053 (*y* ^−1^)	[50]
*μ*_*i*_	Natural birth and death (per capita)	0.0104 - 0.0143 (*y* ^−1^)	[51]
*ρ*	Relapse (per capita)	0.0010 - 0.0083 (*y* ^−1^)	[52, 52, 53]
*γ*_*i*_	Activation from latency in Patch *i* (per capita)	0.0017 - 0.0036 (*y* ^−1^)	[28]
*q*	Proportion of individuals that develop latent TB	0.9 (dimensionless)	[51]
*p*_*ij*_	Proportion of time that residents of Patch *i* spend in Patch *j*	Varies (dimensionless)	–

## Results

### Model analysis

The disease-free equilibrium of (6) is located at the origin of the positive orthan $\mathbb {R}^{4}_{+}$, that is $E_{0}=0_{\mathbb {R}^{4}_{+}}$. The basic reproduction number ($\mathcal R_{0}$) of Model (6) is computed following the next generation method [39, 40]. We decompose System (6) into a sum of the “new infection” vector, denoted by $\mathcal F$, and the “transition” vector, denoted by $\mathcal {V}$. Hence,

$$ \begin{aligned} \left[\begin{array}{l} {\dot{L}}_{1} \\ {\dot{L}}_{2}\\ {\dot{E}}_{1} \\ {\dot{E}}_{2} \end{array}\right] & = \mathcal{F}+\mathcal{V} \\ & = \left[\begin{array}{l} q\sum_{j=1}^{2}\beta_{j} p_{1j}(N_{1}-L_{1}-I_{1})\frac{\sum_{k=1}^{2}p_{kj}I_{k}}{ \sum_{k=1}^{2}p_{kj}N_{k}}\\ q\sum_{j=1}^{2}\beta_{j} p_{2j}(N_{2}-L_{2}-I_{2})\frac{\sum_{k=1}^{2}p_{kj}I_{k}}{ \sum_{k=1}^{2}p_{kj}N_{k}}\\ (1-q)\sum_{j=1}^{2}\beta_{j} p_{1j}(N_{1}-L_{1}-I_{1})\frac{\sum_{k=1}^{2}p_{kj}I_{k}}{ \sum_{k=1}^{2}p_{kj}N_{k}}\\ (1-q)\sum_{j=1}^{2}\beta_{j} p_{2j}(N_{2}-L_{2}-I_{2})\frac{\sum_{k=1}^{2}p_{kj}I_{k}}{ \sum_{k=1}^{2}p_{kj}N_{k}} \end{array}\right] + \\ & \quad + \left[ \begin{array}{l} -\sum_{j=1}^{2}\delta_{j} p_{1j}L_{1}\frac{\sum_{k=1}^{2}p_{kj}I_{k}}{ \sum_{k=1}^{2}p_{kj}N_{k}}-(\gamma_{1}+\mu_{1})L_{1}+\rho_{1} I_{1}\\ -\sum_{j=1}^{2}\delta_{j} p_{2j}L_{2}\frac{\sum_{k=1}^{2}p_{kj}I_{k}}{ \sum_{k=1}^{2}p_{kj}N_{k}}-(\gamma_{2}+\mu_{2})L_{2}+\rho_{2} I_{2}\\ \sum_{j=1}^{2}p_{1j}\delta_{j} L_{1}\frac{\sum_{k=1}^{2}p_{kj}I_{k}}{ \sum_{k=1}^{2}p_{kj}N_{k}}+\gamma L_{1}-(\mu_{1}+\rho_{1}) I_{1}\\ \sum_{j=1}^{2}p_{2j}\delta_{j} L_{2}\frac{\sum_{k=1}^{2}p_{kj}I_{k}}{ \sum_{k=1}^{2}p_{kj}N_{k}}+\gamma L_{2}-(\mu_{2}+\rho_{2}) I_{2} \end{array}\right] \end{aligned}  $$

The rationale behind the presence of nonlinear terms, which represent the infectiousness of latent by infectious individuals, in the $\mathcal {V}$ vector is that these terms do not, technically, represent “new infection”. By denoting *F* and *V*, the Jacobian matrices of $\mathcal {F}$ and $\mathcal {V}$ respectively, evaluated at the disease free equilibrium *E*_0_, the basic reproduction number is the spectral radius of the next generation matrix −*F* *V* ^−1^ [39, 40]. Hence, $\mathcal R_{0}=\rho (-FV^{-1})$ where

$$-FV^{-1}= \left[\begin{array}{cccc} q\gamma_{1}k_{11} & q\gamma_{2}k_{12} & q(\mu_{1}+\gamma_{1})k_{11} & q(\mu_{2}+\gamma_{2})k_{21} \\ q\gamma_{1}k_{21}& q\gamma_{2}k_{22} & q(\mu_{1}+\gamma_{1})k_{21} & q(\mu_{2}+\gamma_{2})k_{22}\\ (1-q)\gamma_{1}k_{11} & (1-q)\gamma_{2}k_{12} & (1-q)(\mu_{1}+\gamma_{1})k_{11} & (1-q)(\mu_{2}+\gamma_{2})k_{12} \\ (1-q)\gamma_{1}k_{21}& (1-q)\gamma_{2}k_{22} & (1-q)(\mu_{1}+\gamma_{1})k_{21} & (1-q)(\mu_{2}+\gamma_{2})k_{22} \end{array}\right] $$

where

$$\begin{aligned} k_{11} &=\left(\frac{\beta_{1}p^{2}_{11}N_{1}}{p_{11}N_{1}+p_{21}N_{2}}+\frac{\beta_{2}p^{2}_{12}N_{1}}{p_{12}N_{1}+p_{22}N_{2}}\right)\frac{1}{\mu_{1}(\gamma_{1}+\mu_{1}+\rho_{1})}\\ &=\left(\frac{\beta_{1}p^{2}_{11}N_{1}}{p_{11}N_{1}+p_{21}N_{2}}+\frac{\beta_{2}p^{2}_{12}N_{1}}{p_{12}N_{1}+p_{22}N_{2}}\right)\frac{\mathcal{R}_{0}^{1}}{\beta_{1}(\gamma_{1}+(1-q)\mu_{1})}, \end{aligned} $$

$$\begin{aligned} k_{12} &=\left(\frac{\beta_{1}p_{11}p_{21}N_{1}}{p_{11}N_{1}+p_{21}N_{2}}+\frac{\beta_{2}p_{12}p_{22}N_{1}}{p_{12}N_{1}+p_{22}N_{2}}\right)\frac{1}{\mu_{2}(\gamma_{2}+\mu_{2}+\rho_{2})}\\ &=\left(\frac{\beta_{1}p_{11}p_{21}N_{1}}{p_{11}N_{1}+p_{21}N_{2}}+\frac{\beta_{2}p_{12}p_{22}N_{1}}{p_{12}N_{1}+p_{22}N_{2}}\right)\frac{\mathcal{R}_{0}^{2}}{\beta_{2}(\gamma_{2}+(1-q)\mu_{2})}, \end{aligned} $$

$$\begin{aligned} k_{21} &=\left(\frac{\beta_{1}p_{11}p_{21}N_{2}}{p_{11}N_{1}+p_{21}N_{2}}+\frac{\beta_{2}p_{12}p_{22}N_{2}}{p_{12}N_{1}+p_{22}N_{2}}\right)\frac{1}{\mu_{1}(\gamma_{1}+\mu_{1}+\rho_{1})}\\ &=\left(\frac{\beta_{1}p_{11}p_{21}N_{2}}{p_{11}N_{1}+p_{21}N_{2}}+\frac{\beta_{2}p_{12}p_{22}N_{2}}{p_{12}N_{1}+p_{22}N_{2}}\right)\frac{\mathcal{R}_{0}^{1}}{\beta_{1}(\gamma_{1}+(1-q)\mu_{1})}, \end{aligned} $$

and

$$\begin{aligned} k_{22} &=\left(\frac{\beta_{1}p_{21}^{2}N_{2}}{p_{11}N_{1}+p_{21}N_{2}}+\frac{\beta_{2}p_{22}^{2}N_{2}}{p_{12}N_{1}+p_{22}N_{2}}\right)\frac{1}{\mu_{2}(\gamma_{2}+\mu_{2}+\rho_{2})}\\ &=\left(\frac{\beta_{1}p_{21}^{2}N_{2}}{p_{11}N_{1}+p_{21}N_{2}}+\frac{\beta_{2}p_{22}^{2}N_{2}}{p_{12}N_{1}+p_{22}N_{2}}\right)\frac{\mathcal{R}_{0}^{2}}{\beta_{2}(\gamma_{2}+(1-q)\mu_{2})}. \end{aligned} $$

Note that $\mathcal {R}_{0}=f(\mathbb {P},\mathcal {R}_{0}^{1},\mathcal {R}_{0}^{2})$ where $\mathcal {R}_{0}^{1}$ and $\mathcal {R}_{0}^{2}$ are the basic reproductive numbers of patch 1 and 2, respectively, when *p*_11_=1=*p*_22_, that is, when there is no movement. $\mathbb {P} = (p_{ij})_{1\leq i,j\leq 2}$ is referred as the residence times matrix of the model. The corresponding expressions of $\mathcal R_{0}^{1}$ and $\mathcal R_{0}^{2}$ are given by (1).

The analysis of Model (6) suggests that the disease dies out from both patches if $\mathcal R_{0}\leq 1$ or persists in both patches otherwise for the case when *q*=1 and *δ*=0 (i.e., in the absence of fast progression and exogenous infections because the residence times matrix becomes irreducible) (See [41–43] for the mathematical proofs). By assuming *q*=1 through out this study and *δ*>0, numerical simulations suggest complex dynamics (i.e., multiple non-trivial equilibria) for the system.

**Fig. 3:**
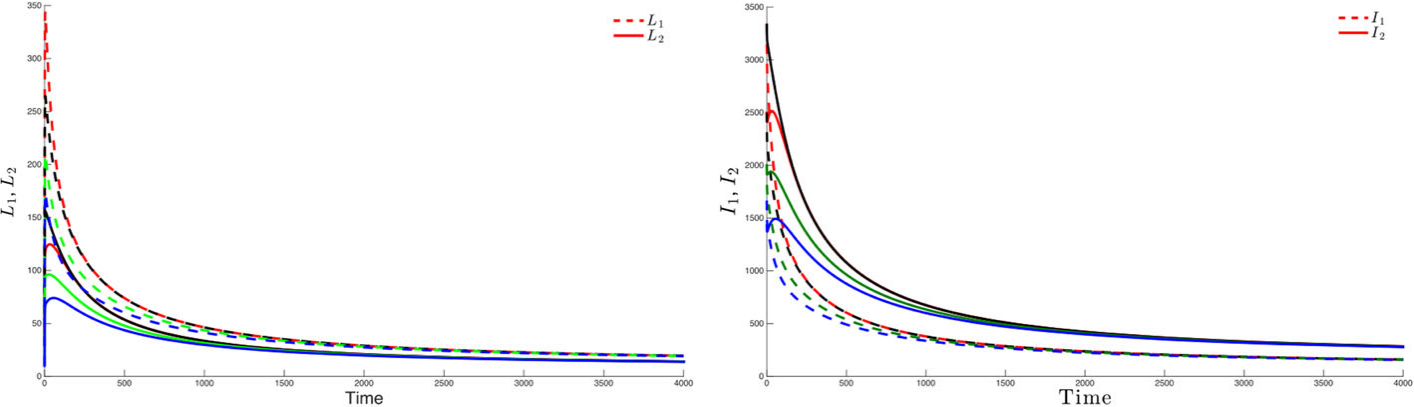
Dynamics of infectious and latent when the two patches are strongly connected and $\mathcal R_{0}>1$. For four different initial conditions, the latent (*top*) and infected (*bottom*) populations of Patch 1 and Patch 2 attain an endemic level if $\mathcal R_{0}>1$

**Fig. 4:**
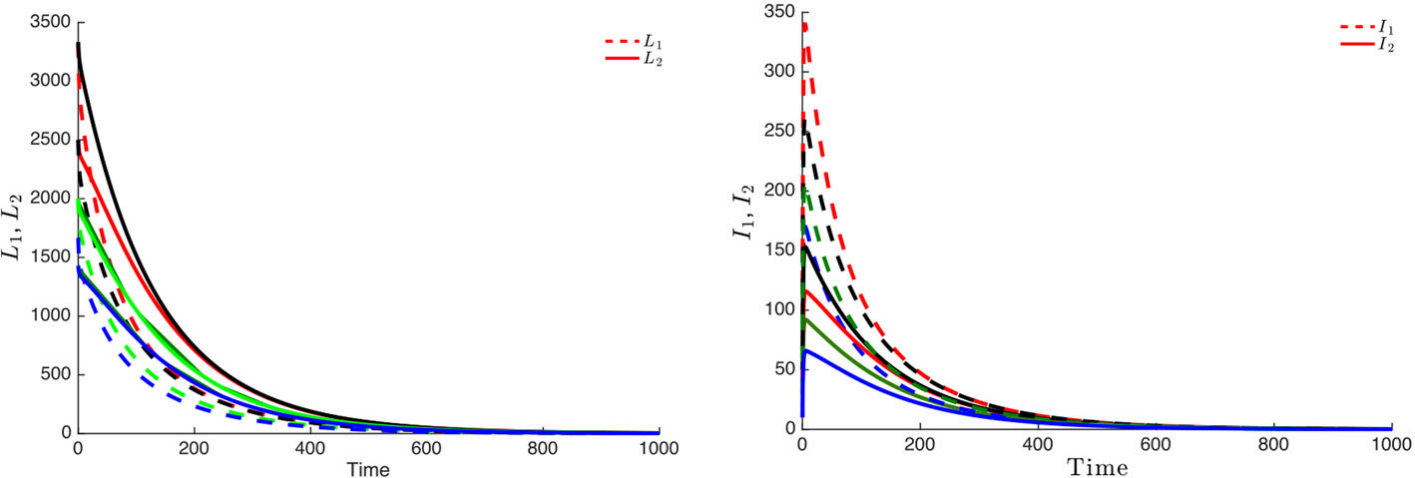
The infectious and latent populations in the two patches converge to zero for four different initial conditions if $\mathcal R_{0}\leq 1$

Figure 3 highlights this robustness, that is for four different initial conditions, the trajectories of the latently infected individuals (Fig. 3 left) as well as the actively-infected (Fig. 3 right) converge towards the endemic state as time becomes large. The case when $\mathcal {R}_{0}\leq 1$, leads to the elimination of the disease from both patches irrespective of the initial conditions as shown in Fig. 4.

**Fig. 5:**
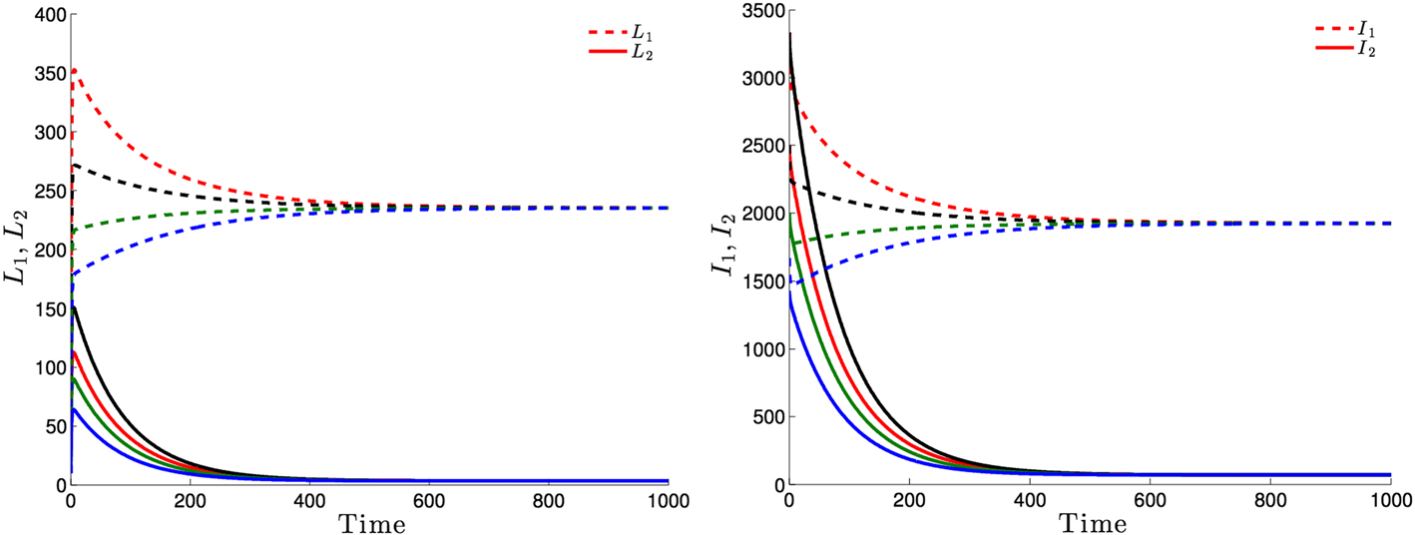
Dynamics when the two patches are weakly connected and $\mathcal R_{0}>1$. The latent (*top*) and infected (*bottom*) of both patches reach an endemic level but Patch 2 approaches a lower level of endemicity ($\mathcal R_{0}^{1}=1.4150$ and $\mathcal R_{0}^{2}=0.1417$ if completely isolated

If Patch 1 is high risk (that is, $\mathcal {R}_{0}>1$) and, if the connectivity between the two patches is not strong (*p*_21_≈0 and *p*_12_≈0), then the disease will persist in both patches, even though that the number of latently-infected and actively-infectious individuals in Patch 2 is small (See Fig. 5 left and right).

**Fig. 6:**
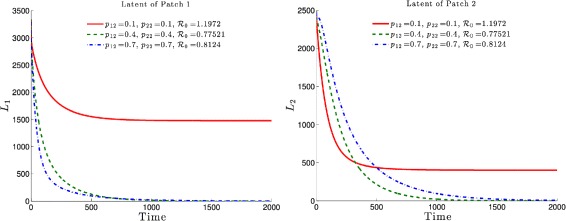
Effects of the residence time matrix on the basic reproduction number and the disease dynamics. In both patches, the *latent* TB populations go to zero if $\mathcal {R}_{0}(\mathbb {P})<1$ and reach an endemic level if $\mathcal R_{0}(\mathbb {P})>1$

**Fig. 7:**
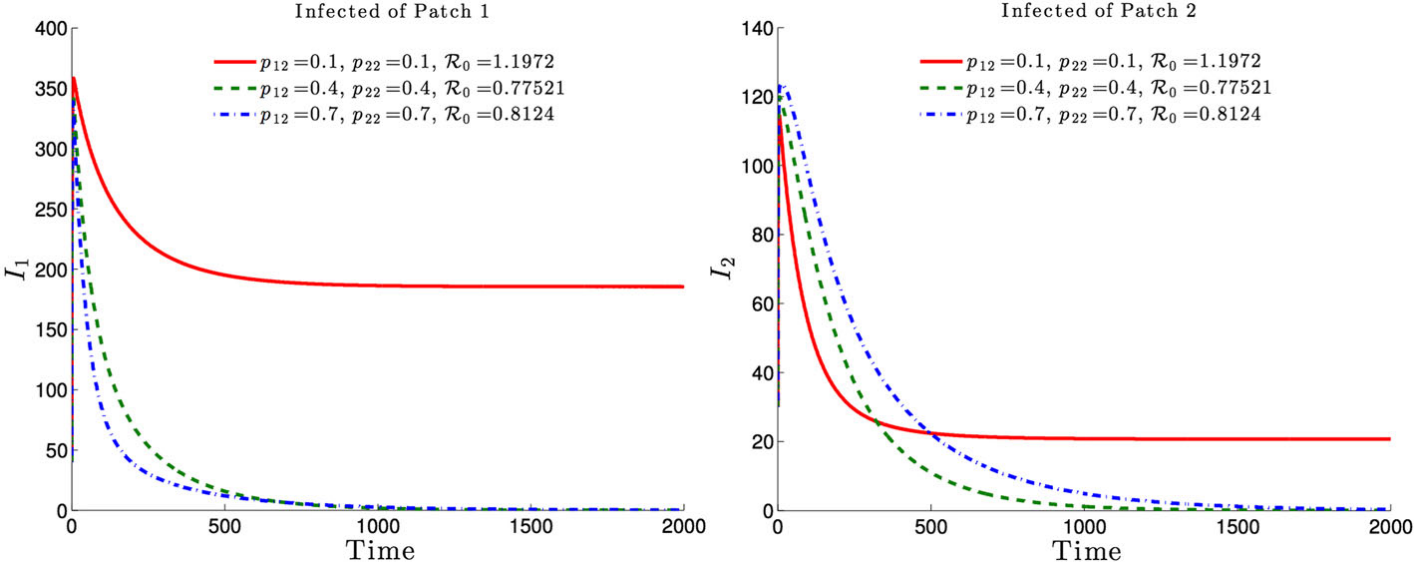
Effects of the residence time matrix on the basic reproduction number and the disease dynamics. In both patches, the *infected* TB populations go to zero if $\mathcal {R}_{0}(\mathbb P)<1$ and reach an endemic level if $\mathcal R_{0}(\mathbb {P})>1$

The effects of the residence times matrix $\mathbb P = (p_{ij})_{1\leq i,j\leq 2}$ on the basic reproduction number ${\mathcal {R}}_{0}(\mathbb {P})$ and, consequently on the disease dynamics, are highlighted in Figs. 6 and 7. It is observed that the basic reproduction number is a decreasing function of *p*_12_, i.e. the residence time of high risk residents (Patch 1 residents) in the low risk Patch 2. Such a decrease would ultimately drive the basic reproduction number to a value less than one with the latent and infected populations, under such mobility schedules, going to zero in both patches (See Figs. 6 and 7, dash-dotted green and dashed blue).

Now, we address the role of mobility, risk and health disparities on TB prevalence levels in a two patch setting. In the next section, we explore the role of the parameters defining mobility, risk and health disparities, on the dynamics of TB.

### The role of risk and mobility on TB prevalence

We now highlight the dynamics of tuberculosis within a two patch system, described by Model (6), under various residence times schemes via numerical experiments. These numerical experiments were carried out using the two-patch Lagrangian modeling framework on pre-constructed scenarios. In particular, we assume that one of the two regions (say, Patch 1) has high TB prevalence. Notice that while the scenarios simulated might be representative of certain regions, we do not model specific cities or regions. Nomenclature of some terms and scenarios are defined in the Table 1.

The interconnection of the two idealized patches demands that individuals from Patch 1 travel to the “safer” Patch 2 to work, to school or for other social activities. It is assumed that the proportion of time that Patch 2-residents spend in Patch 1 is negligible.

In this study we define “high risk” based on the value of the probability of developing active TB using two distinct definitions. In Results section, a high risk patch is defined by having higher direct first time transmission rate (that is *β*_1_>*β*_2_ and *δ*_1_=*δ*_2_). In Results section, a high risk patch is determined by a higher exogenous reinfection rate (or *δ*_1_>*δ*_2_ and *β*_1_=*β*_2_). In addition, in order to explore the role of mobility in different scenarios with population size heterogeneity among the two patches, diverse scenarios are build up by changing the *N*_1_/*N*_2_ ratio. Particularly, we assume that Patch 1 is the denser patch while Patch 2 is assumed to be less dense, that is $\frac {1}{2}N_{1}$ and $\frac {1}{4}N_{1}$. In consequence, contact rates are higher in Patch 1 as compared to corresponding rates in Patch 2.

#### The role of risk as defined by direct first time transmission rates

In this subsection, we explore the impact of heterogeneity in direct first time transmission rates between patches. Assuming Patch 1 is high risk ($\mathcal {R}_{0}^{1}>1$; obtained by assuming *β*_1_>*β*_2_), while Patch 2, in the absence of visitors would be unable to sustain an epidemic ($\mathcal {R}_{0}^{2}<1$). In addition, the effect of different population ratios (*N*_1_/*N*_2_) is explored.

**Fig. 8:**
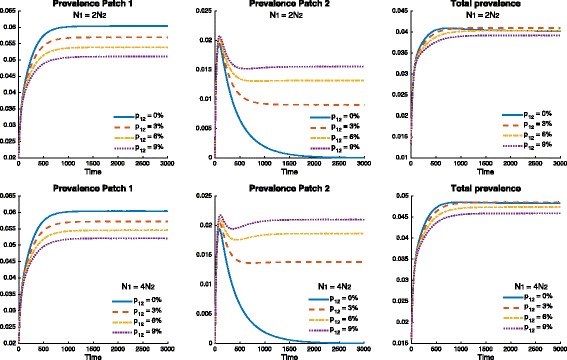
Effect of mobility when *p*_12_=0,3,6 and 9%, for different transmission rates 0.13=*β*_1_>*β*_2_=0.07 (which gives $\mathcal {R}_{0}^{1}=1.5, \mathcal {R}_{0}^{2}=0.8$) and *δ*_1_=*δ*_2_=0.0026, on the prevalence of TB over time. The cumulative prevalence and prevalence for each patch using the following population size proportions $N_{2}=\frac {1}{2}N_{1}$ (*top* figure) and $N_{2}=\frac {1}{4}N_{1}$ (*bottom* figure) are shown as well

Figure 8 shows similar but opposite effects on patch prevalence for Patch 1 and Patch 2 when different residency times (mobility values) are explored (0,3,6 and 9%). Nonetheless, Fig. 8 shows the existence of mobility values (*p*_12_), capable of reducing the overall prevalence of the two patch system. Furthermore, it is observed that different population densities have a noticeable effect on these mobility values.

*These results suggest that increments in mobility, from Patch 1 to Patch 2, reduce TB prevalence in Patch 1 while increasing it in Patch 2. However, the number of total infected individuals from both patches slightly decreases for certain mobility patterns, a global beneficial effect*.

**Fig. 9:**
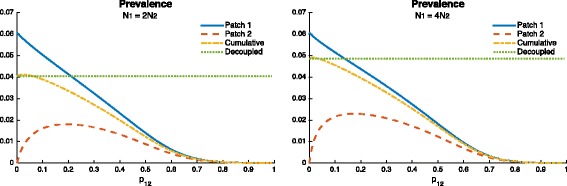
Effect of mobility in the case of different transmission rates 0.13=*β*_1_>*β*_2_=0.07 (which gives $\mathcal {R}_{0}^{1}=1.5, \mathcal {R}_{0}^{2}=0.8$) and *δ*_1_=*δ*_2_=0.0026, on the endemic prevalence using two different population size proportions $N_{2}=\frac {1}{2}N_{1}$ (*left* figure) and $N_{2}=\frac {1}{4}N_{1}$ (*right* figure). The *green horizontal doted line* represents the decoupled case (i.e., the case when there is no movement between patches)

In Fig. 9, we can observe how mobility values *p*_12_ impact prevalence at both the patch and at the system level. At the individual patch level, we have the same trends as in Fig. 8, but now we can observe the existence of a threshold value *p*_12_ (see Red and yellow curve in Fig 9a), for which mobility is always beneficial. That is, completely cordoning off infected regions may not be a good idea to control TB. On the other hand, as long as the mobility value *p*_12_ between high risk and low-risk regions is maintained above the critical value, mobility might become an important factor to control TB outbreaks.

*Furthermore, it is possible that when Patch 1 (riskier patch) has a bigger population size, mobility may turn out to be beneficial; the higher the ratio in population sizes, the higher the range of beneficial “traveling” times (* *p*_12_ *)*.

#### The impact of risk as defined by exogenous reinfection rates

Similarly, focusing on the impact exogenous reinfection has on the TB transmission dynamics, we assume that direct first time transmission rates are the same in both patches (*β*_1_=*β*_2_). In addition, we assume the disease has reached an endemic state in both patches, that is, $\mathcal {R}_{0}^{1}>1$ and $\mathcal {R}_{0}^{2}>1$. However, Patch 1 remains the riskier, due to the assumption that exogenous reinfection in Patch 1 is higher than in Patch 2 (*δ*_1_>*δ*_2_).

**Fig. 10:**
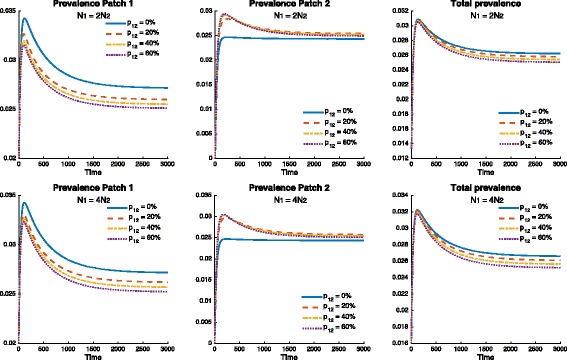
Effect of mobility for *p*_12_=0,20,40 and 60%, when risk is defined by the exogenous reinfection rates 0.0053=*δ*_1_>*δ*_2_=0.0026 and *β*_1_=*β*_2_=0.1 (which gives $\mathcal {R}_{0}^{1}=\mathcal {R}_{0}^{2}=1.155$), on the prevalence over time. The cumulative prevalence and prevalence for each patch are simulated using the following population size proportions $N_{2}=\frac {1}{2}N_{1}$ (*top* figure) and $N_{2}=\frac {1}{4}N_{1}$ (*bottom* figure)

As in the previous case, prevalence levels in Patch 1 are being reduced by mobility (*p*_12_), while prevalence is being increased in Patch 2. Nevertheless, the reduction of prevalence in Patch 1 is greater than the prevalence increment in Patch 2 for most mobility values *p*_12_. Figure 10 suggests the existence of a threshold for which mobility is beneficial for the entire system. Furthermore, the effect of population density can be observed once again favoring higher density heterogeneity between the two patches.

**Fig. 11:**
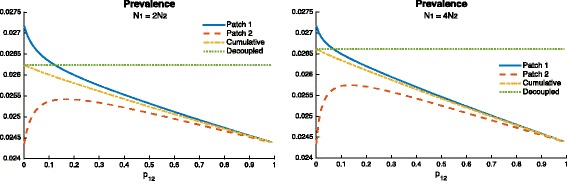
Effect of mobility when risk is defined by the exogenous reinfection rates 0.0053=*δ*_1_>*δ*_2_=0.0026 and *β*_1_=*β*_2_=0.1 (which gives $\mathcal {R}_{0}^{1}=\mathcal {R}_{0}^{2}=1.155$), on the endemic prevalence using two different population size proportions $N_{2}=\frac {1}{2}N_{1}$ (*left* figure) and $N_{2}=\frac {1}{4}N_{1}$ (*right* figure). The *green doted line* represents the decoupled case (i.e., the case when there is no movement between patches)

Figure 11 shows the mobility threshold and how it is impacted by density. This would suggest that mobility between two patches undergoing TB outbreaks with high density heterogeneity (in which the riskier patch is denser) would result in lower TB prevalence levels for the combined system.

*Within this framework, parameters and scenarios, our model suggest that direct first time transmission plays a central role on TB dynamics when mobility is considered. Although mobility also reduces the overall prevalence when exogenous reinfection differs between patches, its impact is small as compared to direct first time transmission results*.

**Fig. 12:**
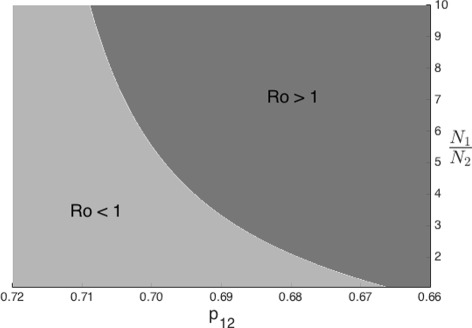
Effect of mobility and population size proportions on the global basic reproductive number $\mathcal {R}_{0}$ when 0.13=*β*_1_>*β*_2_=0.07 and *δ*_1_=*δ*_2_=0.0026

Finally, Fig. 12 shows the relationship between population densities and mobility (*p*_12_) with respect to the basic reproductive number $\mathcal {R}_{0}$. In this case we only explore the first case: direct first time transmission heterogeneity and found out that in this case mobility could indeed eliminate a TB outbreak.

## Discussion

According to the World Health Organization (WHO) [1], in 2014, 80% of the reported TB cases occurred in 22 countries, all developing countries. Efforts to control TB have been successful in many regions of the globe and yet, we still see 1.5 million people die each year. In consequence, TB, faithful to its history [44], still poses one of the greatest challenges to global health. Recent reports suggest that established control measures for TB have not been adequately implemented, particularly in sub-Saharan countries [45, 46]. In Brazil rates have decreased but relapse is more important than reinfection [26, 47]. Finally, in Cape Town, South Africa, a study [48] showed that in high incidence areas, individuals who have received TB treatment and are no longer infectious are at the highest risk of developing TB instead of being the most protected.

Hence, policies that do not account for population specific factors are unlikely to be effective. Without a complete description of the attributes of the community in question, it is almost impossible to implement successful intervention programs that are capable of generating low reinfection rates through multiple pathways and low number of drug resistant cases. Intervention programs must educate populations and their government officials on the benefits, factors, and cost associated with population-based TB prevention and control programs. Intervention must account for the risks that are inherent with high levels of migration as well as with local and regional mobility patterns between areas defined by high differences in TB risk.

In this manuscript, we have focused on the role of ‘daily’ mobility within high and low-risk areas and their potential impact on TB dynamics and control. A situation that is not so uncommon in areas where extreme levels of social, economic and health disparities rule. We carry out the discussion using a simplified framework, that is, a two-patch system, that captures, in a rather ‘dramatic’ way the dynamics between two worlds; the world of the haves and the have nots. The results are highlighted via the simulation of simplified extreme scenarios, as the main objective of this manuscript is to stress the impact of disparities.

As expected, the model analysis suggests that the dynamics of TB depend on the basic reproduction number ($\mathcal {R}_{0}$), which in turn is the function of model parameters that includes direct first transmission and exogenous (reinfection) transmission rates for a single patch system and also includes residency times for a two patch system. The simulations of specific extreme scenarios suggest that short term mobility between heterogeneous patches does not always contributes to overall increases in TB prevalence. The results show that when risk is considered only in terms of exogenous reinfection, the global TB prevalence remains almost unchanged, compared to the effect of direct new infection transmission. In the case of a high risk direct first time transmission, it is observed that mobile populations may pose detrimental effects on the prevalence levels in both environments (patches). Simulations show that when individuals from the risky population spend on average 25% of their time or less in the safer patch the overall prevalence reaches its maximum. However, if they spend more, the overall prevalence decreases. Further, in the absence of exogenous reinfections, the model is robust, that is, the disease dies out or persists based on whether or not the basic $\mathcal {R}_{0}$ is below or above unity, respectively. Although, the role of exogenous reinfection seems not that relevant on overall prevalence, the fact remains that such mode of transmission increases the risk that come from large displacement of individuals, due to catastrophes or conflict, to TB-free areas.

Our ability to interpret information regarding the local origin of mobile individuals accurately would facilitate prompt responses in the face of initiation of an epidemic. During the development and implementation of training and educational programs the necessity to *avoid stigmatizing and further marginalization of groups that may have already experienced some kind of discrimination* is essential to avoid isolation, since it prevents integration, and reduces compliance [49]. A situation that cannot be ignored in today’s world where conflicts have dislocated the lives of millions and generated new migration patterns that includes millions of refugees.

Failure to adequately incorporate and address these challenges may result in considerable delays. As noted in [34], ignoring exogenous reinfections, that is, establishing policies that focus exclusively on the reproductive number $\mathcal {R}_{0}$, would amount to ignoring the role of dramatic changes in initial conditions, now more common than before, due to the displacement of large groups of individuals, the result of catastrophes and conflict.

## Conclusions

This modeling study highlights critical social behaviors mechanisms that can facilitate or eliminate Tuberculosis infection in vulnerable populations. The results suggest that an increase in movement rates between the two distinct risks regions can reduce TB prevalence in a high risk patch (and slightly increase in low risk patch) while decreasing the number of total infected individuals in both patches. That is, when population size heterogeneity between patch 1 and patch 2 is large (*N*_1_>>*N*_2_), mobility from this patch to other low risk patch may provide global benefits in terms of low overall prevalence. Moreover, the higher the ratio in population sizes between distinct risk patches, the larger the benefit under the same “traveling” patterns.

In addition, the direct first time transmission rate plays a central role on TB dynamics when mobility is considered. Mobility also reduces the overall prevalence when exogenous reinfection rates differs between patches, however, its impact on the prevalence is relatively small as compared to the impact of the direct first time transmission rates.

## Abbreviations

HIV: Human immunodeficiency virus
$\mathbb {P}$: Residence times matrix
$\mathcal {R}_{0}$: The basic reproduction number
TB: Tuberculosis
WHO: World health organization’s
